# Psychometric Validation of the Depression, Anxiety and Stress Scale (DASS-21) in Portuguese Youth Transitioning to Higher Education

**DOI:** 10.3390/healthcare14010128

**Published:** 2026-01-04

**Authors:** Luís Loureiro, Ana Teresa Pedreiro, Rosa Simões, Inês Batista, Amorim Rosa, Tânia Morgado

**Affiliations:** 1 Mental Health and Psychiatric Nursing Scientific-Pedagogical Unit, Health Sciences Research Unit: Nursing (UICISA: E), Nursing School of Coimbra, Coimbra University, Avenida Bissaya Barreto s/n, 3004-011 Coimbra, Portugal; rosasimoes@esenfc.pt (R.S.); amorim@esenfc.pt (A.R.); tmorgado@uc.pt (T.M.); 2 School of Economics, Management and Political Science, University of Minho, Campus de Gualtar, 4710-057 Braga, Portugal; teresa.pedreiro@eeg.uminho.pt; 3 Coimbra Local Health Unit, Praceta Professor Mota Pinto, Celas, 3004-561 Coimbra, Portugal; inesbatista93@hotmail.com

**Keywords:** youth, depression, anxiety, stress, confirmatory factor analysis, scale validation

## Abstract

**Background/Objectives**: The transition to higher education is a critical phase of human development that makes adolescents and young adults particularly vulnerable to mental health problems, such as depression, anxiety, and stress. This study aimed to evaluate the psychometric properties of the Portuguese version of the Depression, Anxiety and Stress Scale-21 Items (DASS-21) among first-year undergraduate nursing students. **Methods**: A methodological study was conducted with 225 undergraduate nursing students, aged 17 to 18 years, from a higher education institution in central Portugal. Data were collected using the Google Forms platform. Confirmatory factor analysis was conducted to test three competing models: a single-factor model, a three-factor correlated model, and a second-order factor model. Reliability was assessed using composite reliability, and validity was evaluated using average variance extracted and the Fornell–Larcker criterion for discriminant validity. **Results**: Factor analyses revealed that the three-factor correlated model fit the data best overall, showing superior fit indices compared to the competing models (χ^2^/df = 2.37; CFI = 0.90; and RMSEA = 0.08; TLI = 0.88 and SRMR = 0.04). Composite reliability was high across all tested models, ranging from 0.84 to 0.94. The analysis of score distributions by category revealed a high prevalence of severe or extremely severe symptoms of anxiety, stress, and, to a lesser extent, depression. A statistically significant association was found between higher symptom severity and prior familiarity with mental illness. **Conclusions**: The DASS-21 proved to be a valid and reliable instrument for assessing psychological distress in higher education students. These findings underscore the urgent need for mental health programs in higher education institutions that focus on early detection and intervention, particularly for students initiating their studies and those with a history of mental health problems.

## 1. Introduction

Adolescence is a stage of human development marked by multiple physical, cognitive, emotional, and social changes. During this period, adolescents face transitions related to school, family, and interpersonal relationships, which make them particularly vulnerable to the onset of mental health (MH) problems, such as emotional disorders characterized by symptoms of depression, anxiety, and stress [1,2].

Adolescence is also the time when the transition to higher education usually occurs. This critical and complex period poses significant challenges to the MH and well-being of young students, with potential implications for their academic success, as it encompasses personal, social, and emotional development dimensions [3]. Young people, especially those who move away from home and are physically separated from their families, often find themselves simultaneously facing unprecedented autonomy and responsibilities as they must now manage their entire personal and academic lives.

Various empirical studies have demonstrated the complexity of this transition, highlighting the need for students to develop coping and self-regulation strategies to successfully manage academic and social challenges [4,5].

In this regard, it has been suggested that studies on higher education students’ MH should address not only MH but also well-being. Specifically for MH, it is necessary to assess its most negative dimension (psychological distress), as evidence suggests that students experience high levels of depression, anxiety, and stress [4,6].

Assessing MH with valid and reliable instruments is essential for the early detection of negative emotional symptoms in adolescents and enables the implementation of strategies that promote MH and prevent psychological distress [7,8].

One instrument used to assess emotional states is the Depression, Anxiety and Stress Scale (DASS), developed by Lovibond and Lovibond [9]. The DASS-21, a shorter version of DASS, consists of 21 items distributed across three subscales that assess symptoms of depression, anxiety, and stress, respectively.

The DASS-21 is based on a dimensional approach to psychopathology. Unlike categorical models, it allows for the assessment of symptom severity along a continuum rather than limiting itself to diagnosing the presence or absence of symptoms [9,10]. Factor analyses supported the development of the scale, demonstrating the existence of three defined factors (depression, anxiety, and stress). This is consistent with the basis of the scale, albeit distancing itself from the tripartite model, which proposes a common core of negative affect (distress), as well as specific components for depression (anhedonia) and anxiety (physiological hyperarousal) [11].

In general, the DASS-21 support model and the tripartite model are two fundamental, yet distinct, conceptual frameworks in psychopathology that aim to explain the comorbidity between anxiety and depression. The two models differ in their hierarchical structure and explanatory focus on the factors that constitute them. The authors of the tripartite model [11] start from the close relationship between depression and anxiety, conceptualizing the symptoms specific to depression and anxiety as separate factors while aggregating symptoms shared by both conditions (nonspecific symptoms) into a single factor. Thus, the tripartite model comprises two specific factors (depression and anxiety) and one mixed factor, referred to as the distress factor.

The tripartite model is essentially etiological, aiming to explain why depression and anxiety share many symptoms (i.e., their comorbidity). It posits a common factor, negative affect or general distress, which is represented by the overlapping symptoms of anxiety and depression and reflects a general vulnerability to experiencing negative emotions. In addition, there are two specific factors: physiological hyperarousal/somatic tension, which is primarily associated with anxiety, and low positive affect, which is primarily associated with depression.

As previously mentioned, the DASS-21 is based on a three-factor, first-order structure, measuring three distinct but correlated factors that are treated independently. In the DASS-21, the stress dimension most closely corresponds to the common factor of the tripartite model (negative affect). The anxiety dimension reflects the specific factor of physiological hyperarousal, encompassing physical symptoms, while the depression dimension captures symptoms of loss of pleasure and interest, corresponding to low positive affect [9].

In short, some authors [9,11] have sought to address an empirical finding: the high correlation between anxiety and depression, as well as the difficulty in differentiating between them. While Clark and Watson [11] proposed that negative affect (distress factor) was the common element in anxiety and depression, distinguished by specific factors such as low positive affect in depression and physiological hyperarousal in anxiety, Lovibond and Lovibond [9] agreed that anxiety and depression are distinct constructs and included a third factor: stress.

In terms of evidence, studies using the DASS-21 [12,13,14,15,16,17,18,19] consistently present model solutions that demonstrate the instrument’s good reliability, validity, and stability, with particular emphasis on the three-factor model. The majority of these studies support the three-factor structure (Depression, Anxiety, and Stress) as a valid and replicable solution across different cultures [18,19]. For instance, research conducted in the Portuguese context reported that the three-dimensional structure exhibited good fit indices [12,13]. However, more recent multicultural studies have also found support for a two-factor model, comprising a general factor of psychological distress alongside the specific factors. These findings are particularly evident among university students from multiple countries [15,16,17,18,19].

Therefore, the DASS-21 is an adequate instrument because it demonstrated good psychometric properties in adult and higher education student populations in different cultural contexts, including Portugal [12,13,19,20,21,22].

This study aimed to evaluate the psychometric properties (reliability and validity) of the DASS-21 when applied to first-year nursing students. Three models were evaluated: a single-factor model, a three-factor correlated orthogonal model, and a second-order factor model.

## 2. Materials and Methods

### 2.1. Research Design and Question

This study used a methodological design to test three competing models: the single-factor model, the three-factor correlated model, and the second-order factor model.

Research question: Which of the competing models (single factor, three-factor, or second-order factor) demonstrates the best psychometric properties for the DASS-21?

### 2.2. Settings

This study was conducted at a higher education institution in central Portugal during undergraduate nursing students’ orientation week in September 2024.

### 2.3. Instruments

Sociodemographic questionnaire

The questionnaire included questions about sex, age, parents’ educational attainment, and place of residence.

Depression, Anxiety and Stress Scale-21 (DASS-21)

The DASS-21 is the short form of the DASS-42 [9]. It consists of 21 items across three subscales that assess emotional states: depression, anxiety, and stress. Each subscale comprises seven items. In this study, a version translated and validated for the Portuguese population was used [23].

Depression subscale items:

3. I couldn’t seem to experience any positive feeling at all; 5. I found it difficult to work up the initiative to do things; 10. I felt that I had nothing to look forward to; 13. I felt down-hearted and blue; 16. I was unable to become enthusiastic about anything; 17. I felt I wasn’t worth much as a person; 21. I felt that life was meaningless.

Anxiety subscale items:

2. I was aware of dryness of my mouth; 4. I experienced breathing difficulty (e.g., excessively rapid breathing, breathlessness in the absence of physical exertion); 7. I experienced trembling (e.g., in the hands); 9. I was worried about situations in which I might panic and make a fool of myself; 15. I felt I was close to panic; 19. I was aware of the action of my heart in the absence of physical exertion (e.g., sense of heart rate increase, heart missing a beat); 20. I felt scared without any good reason.

Stress subscale items:

1. I found it hard to wind down; 6. I tended to over-react to situations; 8. I felt that I was using a lot of nervous energy; 11. I found myself getting agitated; 12. I found it difficult to relax; 14. I was intolerant of anything that kept me from getting on with what I was doing; 18. I felt that I was rather touchy.

Respondents indicated how much the statement applied to you over the past week using a four-point Likert-type scale (0 = did not apply to me at all; to 3 = applied to me very much or most of the time).

A question regarding familiarity with mental illness was also included. In this case, students were asked if they had ever received any type of intervention from an MH professional for MH-related issues.

### 2.4. Data Analysis

In this study, data analysis was performed using AMOS software (Version 30; SPSS Inc., Chicago, IL, USA) and IBM SPSS Statistics (Version 30). Appropriate summary statistics (mean and standard deviation) and absolute and percentage frequencies were calculated.

Reliability was assessed using composite reliability (CR) for all tested models, following the recommendations of Marôco [24].

The presence of outliers was examined using Mahalanobis’ squared distance, and the normality of the variables was assessed through Skewness (Sk) and Kurtosis (Ku) coefficients, both at univariate and multivariate levels. Reference thresholds of Sk < 3 and Ku < 10 were used to determine compliance with normality assumptions.

The goodness of fit of the confirmatory factor analysis (CFA) for each model was evaluated using multiple fit indices: chi-square to degrees of freedom ratio (χ^2^/df), comparative fit index (CFI), goodness of fit index (GFI), root mean square error of approximation (RMSEA), Akaike information criterion (AIC),TLI (Tucker–Lewis Index) and Square Root Mean Square Residual (SRMR) [25].

Model adequacy was further assessed through factor loadings, individual item reliability, and modification indices (>11.0 and *p* < 0.001). The following cutoff values were used: χ/df ≤ 3 (good fit), CFI ≥ 0.95 (good fit), GFI ≥ 0.95 (good fit), RMSEA ≤ 0.06 (good fit), TLI ≥ 0.95 (good fit), and SRMR ≤ 0.08 (good fit).

Convergent validity was examined using the average variance extracted (AVE), while discriminant validity was tested following the Fornell–Larcker criterion [26], namely for the two correlated factor solutions [18]. Specifically, the squared correlations between factors were compared with the AVE values corresponding to each factor. As the DASS-21 was the sole instrument used to assess the constructs, and no alternative measures were available for comparison, it was not possible to establish external convergent or discriminant validity.

### 2.5. Data Collection

Data were collected in September 2024, during student orientation week. The questionnaire was administered in the classroom using the Google Forms platform, with the researchers present at each administration session. Access to the questionnaire was provided via a QR code in the classrooms. Participants provided electronic informed consent, confirming that they had understood the study objectives and agreed to participate voluntarily. The average time for completing the questionnaire was 6.17 min, and the response rate was 75.0%.

### 2.6. Ethical Considerations

Both the study and the questionnaire were approved by the institution’s administration and the Ethics Committee of the Health Sciences Research Unit: Nursing (P603/06 2019). The inclusion criteria were to voluntarily agree to participate in the study and to provide electronic consent, when required.

## 3. Results

### 3.1. Sample

The sample consisted of 225 students, with a mean age of 17.74 years (SD = 0.44 years), 83.6% of whom were female. The majority of the sample (172; 76.4%) had moved to a different municipality to attend this higher education institution.

### 3.2. Item Statistics

Based on the descriptive results of the subscale items (Table 1), which assess the severity of depression, anxiety, and stress symptoms, the scores for all items ranged from a minimum of 0 to a maximum of 3, indicating the possible answer range for each item. Item means varied significantly, from 0.33 (item 21) to 1.48 (item 18), suggesting different levels of perceived symptom intensity. The median values for most items were 0 or 1, indicating that most answers scored lower scale values. Sk and Ku values also differed. For example, items 10 (Sk = 1.61; Ku = 1.92) and 17 (Sk = 1.55; Ku = 1.79) showed pronounced skewed distributions, suggesting a greater concentration of answers at lower values with some higher extreme values. Conversely, items such as item 18 (Sk = 0.25; Ku = −0.81) demonstrate a more symmetrical and less skewed distribution, suggesting a greater dispersion of answers across the scale.

### 3.3. Results of Competing Models

The CFA revealed that the single-factor model showed a poorer fit (χ^2^/df = 3.45, CFI = 0.81, and RMSEA = 0.11). The second-order model and the three-factor model provided a better fit than the single-factor model. Although the CFI (0.90) and GFI values (0.85) were good, the RMSEA value of 0.08 indicated an acceptable fit. TLI = 0.88 and SRMR also indicated good fit [24,25].

A comparison of the AIC values indicated that the three-factor model and the second-order model provided the best balance between model fit and parsimony, thus minimizing information loss, followed by the single-factor model (AIC = 736.76).

As was evident from the initial comparison of fit indices, the results confirm that the three-factor model and the second-order model provided a significantly better fit than the single-factor model.

**Figure 1:**
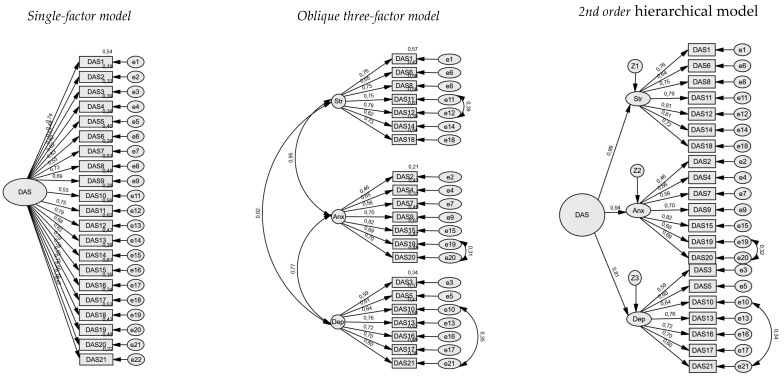
Structural comparison of competing CFA for the DASS-21: single-factor, oblique three-factor, and 2nd order hierarchical models.

Figure 1 presents the factor loadings for each solution: the single-factor model, the three-factor model (Depression, Anxiety, Stress), and the second-order model. Most DASS-21 items had high, statistically significant loadings, suggesting strong measurement of their constructs across all models.

AVE was used to assess convergent validity. As shown in Table 2, for the single-factor (unidimensional) model, the estimated AVE value was 0.42. Although this value can be considered reasonable, it falls below the threshold generally recommended in the literature (AVE ≥ 0.50) [24,25].

After analyzing the modification index values, the initial analysis of the three-factor model revealed significant correlations between the residuals of items 11 (“I felt myself getting agitated”) and 12 (“I found it difficult to relax”) within the stress factor; items 10 (“I felt I had nothing to look forward to”) and 21 (“I felt that life was meaningless”) within the depression factor; and items 19 (“I was aware of the action of my heart in the absence of physical exertion (e.g., sense of heart rate increase, heart missing a beat)”) and 20 (“I felt scared without any good reason”) within the anxiety factor. Residual covariances were incorporated in all cases to account for item-level autocorrelation, which may reflect overlapping semantic content or symptomatic comorbidity.

For the three-factor and second-order models, the AVE values were 0.52 for stress, 0.44 for anxiety, and 0.44 for depression, with the latter two falling below the commonly accepted minimum threshold (AVE ≥ 0.50).

Regarding discriminant validity, the AVE values were compared with the squared correlations between factors. The correlations were as follows: Stress and Anxiety (*r* = 0.95; r^2^ = 0.90), Depression and Stress (*r* = 0.82; r^2^ = 0.72), and Anxiety and Depression (*r* = 0.77; r^2^ = 0.59). Consequently, discriminant validity was not established for all construct pairs in the three-factor correlated model.

High correlations between first-order factors raise questions about the discriminant validity of the second-order factor. In this model, stress, anxiety, and depression serve as general components of the DASS, with very strong loadings on the second-order factor (0.99, 0.94, and 0.81, respectively). This indicates a dominant general factor, consistent with the high second-order loadings, strong inter-factor correlations, and low AVE values for anxiety and depression. Overall, the data from this sample suggest a potential discriminant validity issue. This does not imply that the model is inadequate; rather, it reflects the presence of a strong general factor in the DASS-21—a dominant general factor of psychological distress encompassing three consistent, overlapping dimensions.

In the second-order model, the modification indices suggested including a residual covariance between items 11 and 12. However, the model was deliberately re-specified to exclude this correlation based on the principles of parsimony and construct validity. This approach preserves the integrity of the latent stress construct and supports the validity of the proposed hierarchical structure, providing a more parsimonious solution.

To validate these results, a chi-square difference test was performed between the values of the two best-fitting models: the three-factor correlated model and the second-order model.

χ^2^diff = |χ^2^(three-factor) − χ^2^(second-order)| and *df* = *df*(three-factor) − *df* = *df*(second-order)

χ^2^diff = |434.06 − 462.22|= 28.16 and *df* = 184 − 183 = 1

In this case, the test statistic was χ^2^diff = 28.16 and *df* = 1. According to the chi-square distribution [18] for an alpha of 0.05 and *df* = 1, the theoretical chi-square value was 3.84.

As was evident from the initial comparison of indices, the results confirm that the three-factor model provided a significantly better fit than the second-order factor model.

Despite the theoretical distinction among these constructs in the model proposed by Lovibond and Lovibond [9], the results of this study suggest that, in this sample, the three constructs were not fully independent.

CR values were consistently high across all models (ranging from 0.84 to 0.91), demonstrating excellent internal consistency of the DASS-21 subscales, regardless of the model tested.

Table 3 presents the distribution of Depression, Anxiety, and Stress categories among all students in the sample, varying according to familiarity with mental illness. Familiarity was defined as having previously received treatment from a MH professional for a MH condition.

All associations were statistically significant. In general, students with no previous familiarity with mental illness tended to report normal to moderate symptom intensity, while extreme and extremely severe symptoms were observed among those with prior familiarity with mental illness.

**Figure 2:**
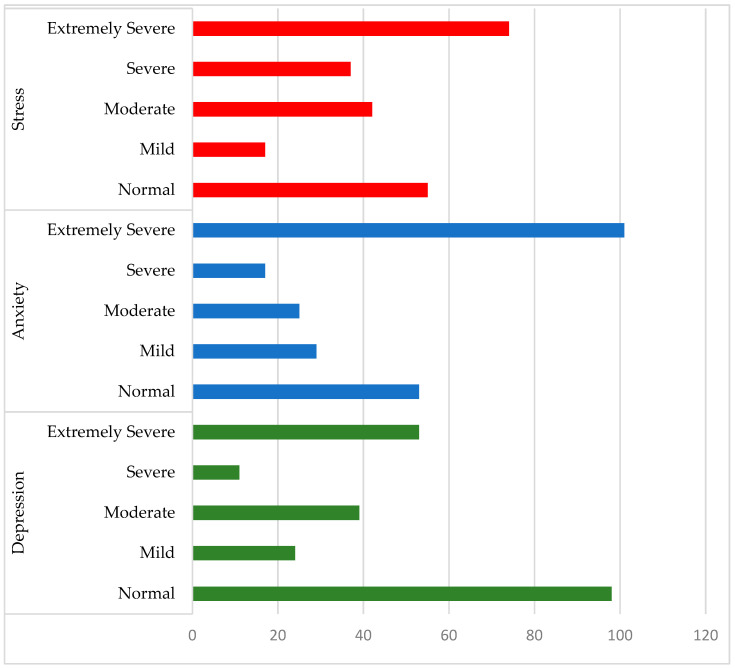
Categories of depression, anxiety, and stress (*N* = 225).

The percentage distributions for the Depression, Anxiety, and Stress categories indicate severe and extremely severe levels, particularly for Stress and Anxiety, whereas these values were comparatively lower for Depression. Figure 2 presents the absolute frequencies of each category across all subscales for all participants.

## 4. Discussion

This study aimed to evaluate the psychometric properties of the DASS-21 in students newly enrolled in the first year of the undergraduate nursing program. This transition to higher education represents a critical period, marked by multiple personal, social, and academic challenges [1,2]. Late adolescence and early adulthood are, consequently, times of particular vulnerability for developing symptoms of psychological distress. Early detection and timely intervention are essential to prevent the worsening of symptoms [3,7,19].

In this context, the CFA indicated that the three-factor correlated model (Depression, Anxiety, and Stress) provided the best fit to the data, with indices slightly higher than those of the second-order model and markedly better than those of the single-factor model. These findings support the structure proposed by Lovibond and Lovibond [9], who conceptualized these dimensions as related yet distinct constructs based on a dimensional approach to psychopathology.

As noted by Henry and Crawford [10], this structure allows not only for the overall assessment of psychological distress but also for the identification of distinct symptomatic profiles, which are fundamental for guiding targeted interventions.

Nevertheless, high correlations were observed between some factors, particularly Anxiety and Stress, limiting the discriminant validity. Similar studies [4,13] point out that, in contexts of high academic pressure, the physiological and cognitive manifestations of anxiety—such as muscle tension, restlessness, and difficulty concentrating—often overlap with stress responses, making empirical distinction between the two constructs challenging.

This overlap can be interpreted in light of the tripartite model [11], which posits a common core of negative affect shared by both Anxiety and Depression, alongside specific components that may not always manifest clearly in young populations and during periods of academic transition.

In summary, although the three-dimensional structure of the DASS-21 provides the best overall fit to the data in this sample, the depression, anxiety and stress factors are not entirely independent. The three-factor model was the most parsimonious, with fit indices superior to those of the single-factor model and slightly better than those of the second-order hierarchical model. However, very high correlations were found between the factors and AVE values were below the recommended level for anxiety and depression, suggesting reduced discriminant validity. On the other hand, the high internal consistency values and strong factor loadings observed point to the presence of an underlying general factor of psychological distress across the three constructs. Therefore, while the three-dimensional solution is statistically the most appropriate, the data indicate that the dimensions of the DASS-21 overlap considerably in this population, reflecting a model in which psychological distress plays a predominant role.

CR was high across all models, ranging from 0.84 to 0.94, indicating excellent internal consistency [24]. These findings reinforce the robustness of the DASS-21 for assessing psychological distress in the Portuguese higher education context. However, the AVE values for Depression and Anxiety were slightly below the recommended threshold of 0.50 [18], suggesting that part of the item variance may be associated with external factors or a general distress construct.

The results of this study are consistent with the international literature, suggesting that although the three-dimensional model of the DASS-21 shows a better fit, there is a strong overlap between the dimensions of depression, anxiety, and stress, pointing to a general factor of psychological distress.

Studies conducted in Portuguese-speaking contexts [12,13,14] confirmed the adequacy of the three-factor model, demonstrating the reliability and validity of its three dimensions. Similar results were observed in cross-cultural studies [18], which verified the stability of the three-factor structure in samples from Australia, China, and Vietnam. These studies also found high correlations between the factors.

Together, these results are consistent with those of the present study in showing that, while the three-factor structure provides the best statistical fit, weak discriminant validity between dimensions appears to be a consistent feature of the DASS-21 across different cultures and contexts [17,18,19]. It should also be noted that some studies consistently validate and support the second-order model [15,16].

The analysis of symptom severity categories revealed worrying data: a substantial proportion of students exhibited severe or extremely severe Anxiety (44%) and Stress (49%), while Depression levels, although lower, remained notable. These figures align with national and international research documenting the high prevalence of psychological distress among higher education students, particularly first-year students [4,6,13]. The higher prevalence of Anxiety and Stress compared to Depression may reflect the situational and reactive nature of these emotional states, which are often triggered by abrupt changes in demands and routines [21].

One relevant finding was the association between familiarity with mental illness and greater symptom severity. This relationship has been observed in previous studies [27]. Therefore, this subgroup of students with experience of MH problems may benefit from closer monitoring and personalized prevention programs [28,29,30].

From an institutional point of view, these results have clear implications. As some authors argue, higher education institutions should invest in well-being promotion strategies that combine primary prevention (e.g., stress management workshops and social-emotional skills training) and early intervention actions (e.g., screening and psychological referral) [2,3]. These measures are particularly relevant in health education, where curricular demands are high and early contact with clinical reality can be an additional stress factor.

Methodologically, this study benefited from an adequate sample size for factor analysis (more than the minimum of 10 subjects per item recommended by some authors [24,31], the use of validated instruments, and data collection during a critical transitional moment. However, the cross-sectional design does not allow for the assessment of the stability of the factor structure over time or capture seasonal variations associated with the academic calendar. In addition, the sample was restricted to a single institution and program and consisted mostly of female students (83.6%), so generalization of the results to other contexts should be done with caution.

## 5. Conclusions

This study reveals that the three-factor DASS-21 model is valid and reliable for assessing symptoms of depression, anxiety, and stress. This model best fits the data. This finding supports the notion that, while these factors are distinct, they are also interconnected. Nevertheless, high correlations were observed between some factors, particularly anxiety and stress, which compromised the discriminant validity. Similar studies suggest that, in contexts of high academic pressure, the physiological and cognitive manifestations of anxiety, such as muscle tension, restlessness, and difficulty concentrating, often overlap with stress responses. For this reason, the empirical distinction between the two constructs is empirically challenging.

Although the three-factor model presents a better statistical fit, we can say that the hierarchical model allows for a more parsimonious and theoretically consistent interpretation of the latent structure. From a conceptual and interpretative point of view, it may be more coherent, as it explains the overlap of factors at the level of a general distress factor.

Notably, the study found a high prevalence of individuals experiencing severe and extremely severe levels of anxiety, stress, and even depression. These findings underscore the need for ongoing student follow-up, with particular attention to their experiences and emotional states. Early detection of severe and extremely severe symptoms, combined with institutional psychological support policies, may help mitigate the impact of distress on the MH and academic success of these young people. These findings also highlight the importance of intervention during the transition to higher education.

From a methodological perspective, the cross-sectional design does not allow for the assessment of the stability of the factor structure over time or the capture of seasonal variations associated with the academic calendar. In addition, the sample was restricted to a single institution and program, consisting mostly of female students (83.6%). Therefore, the generalization of the results to other contexts should be done with caution. Future studies should adopt longitudinal designs, include more heterogeneous samples, and explore the DASS-21 sensitivity to interventions and its predictive capacity in relation to academic and well-being indicators.

## Figures and Tables

**Table 1:** Descriptive statistics for the DASS-21 items (*N* = 225).

Items	Minimum	Maximum	*M*	*SD*	*Md*	*IQR*	*Sk*	*Ku*
DASS-1	0	3	1.02	0.91	1.00	2.00	0.64	−0.35
DASS-2	0	3	0.81	0.86	1.00	1.00	0.84	−0.02
DASS-3	0	3	0.48	0.63	0.00	1.00	1.07	0.58
DASS-4	0	3	0.53	0.77	0.00	1.00	1.39	1.27
DASS-5	0	3	0.97	0.79	1.00	1.00	0.50	−0.19
DASS-6	0	3	0.96	0.90	1.00	2.00	0.63	−0.45
DASS-7	0	3	0.74	0.91	0.00	1.00	1.06	0.17
DASS-8	0	3	1.02	0.87	1.00	2.00	0.45	−0.59
DASS-9	0	3	0.82	0.91	1.00	1.00	0.82	−0.34
DASS-10	0	3	0.52	0.82	0.00	1.00	1.61	1.92
DASS-11	0	3	0.99	0.89	1.00	2.00	0.55	−0.52
DASS-12	0	3	1.16	0.94	1.00	2.00	0.40	−0.73
DASS-13	0	3	0.90	0.86	1.00	1.00	0.65	−0.33
DASS-14	0	3	0.52	0.76	0.00	1.00	1.37	1.18
DASS-15	0	3	0.61	0.84	0.00	1.00	1.19	0.48
DASS-16	0	3	0.48	0.64	0.00	1.00	1.21	1.21
DASS-17	0	3	0.43	0.69	0.00	1.00	1.55	1.79
DASS-18	0	3	0.48	0.92	1.00	1.00	0.25	−0.81
DASS-19	0	3	0.83	0.96	1.00	1.00	0.84	−0.45
DASS-20	0	3	0.67	0.89	0.00	1.00	1.23	0.62
DASS-21	0	3	0.33	0.68	0.00	0.00	1.10	1.80

M = Mean; SD = Standard deviation; Md = Median; IQR = Interquartile Range; Skewness; Ku = Kurtosis.

**Table 2:** Summary of fit statistics for the CFA models tested.

Models	χ^2^	*df*	χ^2^/*df*	CFI	RMSEA	AIC	GFI	TLI	SRMR	AVE	CR
Single-factor	652.76	189	3.45	0.81	0.11	736.76	0.76	0.79	0.05	0.42	0.94
Three-factor	434.06	183	2.37	0.90	0.08	530.06	0.85	0.88	0.04	0.44–0.52	0.84–0.88
2nd order factor	462.22	184	2.51	0.89	0.08	556.23	0.84	0.87	0.04	0.44–0.52	0.87–0.91

**Table 3:** Association of familiarity with mental illness across categories of depression, anxiety, and stress (*N* = 225).

Categories [Depression]	Familiarity with MI		Total	χ^2^
	*No*	*Yes*		
Normal	61 (62.2)	37 (37.8)	98 (43.6)	10.828 * (CV = 0.22)
Mild	17 (70.8)	7 (29.2)	24 (10.7)	
Moderate	21 (53.8)	18 (46.2)	39 (17.3)	
Severe	3 (27.3)	8 (72.7)	11 (4.9)	
Extremely Severe	23 (43.4)	30 (56.6)	53 (23.6)	
Categories [Anxiety]				
Normal	42 (79.2)	11 (20.8)	53 (23.6)	25.974 *** (CV = 0.34)
Mild	20 (69.0)	9 (31.0)	29 (12.9)	
Moderate	14 (56.0)	11 (44.0)	25 (11.1)	
Severe	10 (58.8)	7 (41.2)	17 (7.6)	
Extremely Severe	39 (38.6)	62 (61.4)	101 (44.9)	
Categories [Stress]				
Normal	44 (80.0)	11 (20.0)	55 (24.4)	26.033 *** (CV = 0.34)
Mild	11 (64.7)	6 (35.3)	17 (7.6)	
Moderate	26 (61.9)	16 (38.1)	42 (18.7)	
Severe	14 (37.8)	23 (62.2)	37 (16.4)	
Extremely Severe	30 (40.5)	44 (59.5)	74 (32.9)	
Total	125 (55.6)	100 (44.4)	225	

* *p* < 0.05; *** *p* < 0.001; CV = Cramer’s V.

## Data Availability

The data generated in this study are available on request from the corresponding author due to ethical considerations, including the protection of participants’ privacy and the prevention of potential misuse outside the proper scientific or ethical context.

## Abbreviations

The following abbreviations are used in this manuscript:

AIC	Akaike information criterion
AVE	Average variance extracted
CFA	Confirmatory factor analysis
CFI	Comparative fit index
CR	Composite reliability
CV	Cramer’s V
Df	Degrees of freedom
DASS	Depression, Anxiety and Stress Scale
IQR	Interquartile Range
GFI	Goodness of fit index
Ku	Kurtosis
M	Mean
Md	Median
MH	Mental health
RMSEA	Root mean square error of approximation
SD	Standard deviation
Sk	Skewness
SRMR	Square Root Mean Square Residual
TLI	Tucker–Lewis Index
